# Coordination and Cooperation are Essential: A Call for a Global Network to Enhance Integrated Human Health Risk Resilience Based on China’s COVID-19 Pandemic Coping Practice

**DOI:** 10.1007/s13753-021-00364-4

**Published:** 2021-07-27

**Authors:** Yelin Sun, Tian Liu, Tao Ye, Peijun Shi

**Affiliations:** 1 State Key Laboratory of Earth Surface Processes and Resource Ecology, Beijing Normal University, Beijing, 100875 China; 2 Academy of Plateau Science and Sustainability, Qinghai Normal University, Xining, 810008 China; 3 Faculty of Geographical Science, Beijing Normal University, Beijing, 100875 China

**Keywords:** COVID-19, Human-to-frozen goods-to-human transmission, Government response, Medical resources, Coordination and cooperation

## Abstract

More than a year after its appearance and still rampant around the world, the COVID-19 pandemic has highlighted tragically how poorly the world is prepared to handle systemic risks in an increasingly hyper-connected global social-ecological system. The absence or clear inadequacy of global governance arrangements and mechanisms is painfully distinct and obvious. In this short article, we summarize a set of COVID-19 pandemic-related analyses and lessons that are inspired by Chinese practice. First, strong government response is one of the most important methods to control a pandemic. Second, countries should be concerned about human-to-frozen goods-to-human transmission. Third, sharing resources and experiences through cooperation is crucial to ensure an adequate health response. Based on these insights, we stress the critical importance of coordination and cooperation, and call for a global network to enhance integrated human health risk resilience.

## Introduction

COVID-19 is still spreading rapidly around the world. According to the World Health Organization, a total of 167,011,807 COVID-19 infections and 3,472,068 deaths were reported over 200 countries as of 25 May 2021 (WHO 2021). The pandemic has already caused a major global economic downturn (Albu et al. 2020) and trade between countries also declined (Vidya and Prabheesh 2020).

Compared to the rest of the world, China’s course of combating the COVID-19 pandemic is rather unique. As of 25 May 2021, the total reported infected number of cases in the Chinese mainland is 91,019 of which about 91.1% occurred before April 2020. By 25 May 2021, the total number of reported COVID-19 deaths in China’s mainland was 4,636; about 99.9% of these fatalities occurred before April 2020. In other words, since April 2020, China has seen an effective overall control of COVID-19 spread nationwide, with only local cases occurring from time to time. The main channel of transmission has also seen a shift from communal to “importing” associated with cross-border flows of both people and goods.

From a disaster risk reduction and management perspective, what can we learn from China’s experience that can be instrumental for others who are still battling with the spread of COVID-19? Perhaps more importantly, what are the key insights we can draw that would support the development of a resilient human health system globally in the long term?

Guided by these broad questions, we examined China’s COVID-19 spread and control from the very beginning. In this short article, we focus on learning from three aspects of the Chinese pandemic experience—the role of the government in terms of response measures; the changing transmission channels to highlight the often overlooked human-to-frozen goods-to-human transmission linkages; and the impacts of medical resources.

The rationale for choosing these key aspects to focus upon is, first, that the effective control of virus spread in China has much to do with strong, government-led responses and societal organizational capacity (Han et al. 2020; Liu, Zhang, et al. 2020). Experience in China has shown that a strict lockdown policy can cut down interhuman physical contact and control the pandemic. The critical role of other non-pharmaceutical interventions (for example, restrictions on gathering size) also has been confirmed (Betsch et al. 2020; Cowling et al. 2020; Hsiang et al. 2020). Imposing restrictions in a timely manner can effectively reduce the number of confirmed COVID-19 cases (Bai et al. 2020). Few studies, however, have explored the impact of government response on the COVID-19 pandemic at a global scale.

Second, once communal or person to person transmission is under control, other channels of transmission require much more attention. After April 2020, the main channel of transmission in China shifted to “importation” associated with cross-border flows of people and goods. For example, the COVID-19 outbreak in Beijing between 11 June and 6 August 2020 was traced back to infection through cold-chain logistics from abroad and then spread from person to person internally (Pang et al. 2020). This channel of transmission has not received much attention globally.

Third, not surprisingly, the availability of adequate medical resources is critical to reduction of the case fatality rate of COVID-19. Yet, the rapid outbreak through communal transmissions could quickly overwhelm the existing local or even regional healthcare capacities. This was clearly the case in Wuhan in early 2020, where the case fatality rate in the early stage of the COVID-19 pandemic was high. Therefore, we considered the impact of medical resources on the COVID-19 case fatality rate and examined how to deal with an inadequacy of healthcare systems in the crisis stage of the pandemic.

In the reminder of this article, we first provide more elaboration on the key lessons from each of these areas before we conclude this summary with a call for a global network to enhance integrated human health risk resilience by building a global community of health for all.

## Coordinated, Swift, and Forceful Government-Led Response is Essential

One of the key insights from the Chinese COVID-19 experience is the need for an effective, swift, and well-coordinated government-led response in controlling the spread of the coronavirus. China offered an extreme case of the forceful implementation of restrictive policies with strong organizational capacity, which not only helped to curb the spread of infection in Wuhan City, but also effectively blocked nationwide transmission in China (Tian et al. 2020). From the moment when the outbreak began to be discovered in late December 2019 to the re-opening of Wuhan on 8 April 2020, over a period of 3 months, China achieved an effective control of the coronavirus spread within its borders (Jin et al. 2020). Once the overall spread of the coronavirus was under control (that is, since April 2020), China built a network-smart platform that can evaluate individual COVID-19 health risks and generate QR codes according to trace-track and contact history. Cities set up local “COVID-19 health kit” mini-programs on WeChat, Alipay, and other commonly used mobile apps. These tools record people’s movement and COVID-19 health risk, and largely enabled rapid and effective responses to deal with local outbreaks from time to time. While similar measures were taken around the world, there are considerable variations in the timing of taking such measures, their coverage, and citizen adherence, as well as in the strength and unity of governance and leadership of multisectoral actions (Tangcharoensathien et al. 2021; Xing and Zhang 2021). The important role of government-led response is further verified through our analysis of the relationship between daily government response and the daily growth rate of new infection cases in 164 countries/regions, which was done using policy data of these countries/regions obtained from the Oxford COVID-19 Government Response Tracker (Hale et al. 2021). This dashboard collects the daily number of confirmed cases and deaths since 22 January 2020 and provides up-to-date policy information about government responses to COVID-19. The dataset contains five indices that represent the various aspects of government response to the COVID-19 pandemic. These indices measure government response, containment and health, stringency, economic support, and legacy stringency.

We conducted a Pearson’s correlation analysis between government response and daily growth rate of new infection cases^1^ of the 164 countries/regions. The government response index from the Oxford COVID-19 Government Response Tracker database covers a set of government-led measures including school closing, workplace closing, canceling public events, restrictions on gathering size, closing public transport, stay at home requirements, restrictions on internal movement, restrictions on international travel, income support, debt/contract relief for households, public information campaigns, testing policy, contact tracing, facial coverings, and vaccination policy (Hale et al. 2021). A high government response index indicates that the intensity of measures taken by the government is high. Daily epidemiological data were obtained from an open-source GitHub project, which gathers daily updates from the John Hopkins University Coronavirus Resource Center (Dong et al. 2020), including daily cumulative confirmed cases in the 164 countries/regions.

As expected, 69% of the 164 countries/regions show a significant negative correlation between daily growth rate and government response index, which confirms the effective role of government-led integrated response in reducing the daily growth rate of infection. We further analyzed the relationship between government response and daily growth rate of infection in China, Japan, the United States, the United Kingdom, Italy, and Brazil and found that all countries demonstrated a similar pattern of relationship. But the lagging time as well as the pace of reduction in new infection case growth rate varies among countries. The Chinese government response index showed a significant increase on 23 January 2020 and since 15 February, the daily infection growth rate began to decrease. In Japan, the government response index increased by 45.56 on 16 April and, after 16 days, the daily infection growth rate began to decrease. The daily infection growth rate in the UK has been less than 0.02 since 1 May 2020, 40 days after the date on which the government response index was above 50. In Italy, the government response index showed an increase on 23 February, and after 33 days the daily infection growth rate decreased. In the United States, 50 days after the response index was above 50, the daily infection growth rate decreased. The government response index was above 50 in Brazil on 22 March, and since then the daily infection growth rate decreased in a fluctuating pattern. While the critical and effective role of government-led measures and response in controlling the spread of the coronavirus is evident from all the regions and countries analyzed, there is little evidence for globally coordinated actions.

## Trade Related Human-to-Frozen Goods-to-Human Transmission Needs Attention

When community transmission of coronavirus was under control, several incidences of local outbreaks in China were traced back to human-to-frozen goods-to-human transmission, that is, the source of the infection came through imported goods—mostly frozen food, hence also the name “cold-chain transmission.” For example, Liu and colleagues isolated SARS-CoV-2 from the imported frozen cod package surface in the outbreak on 24 September 2020 in Qingdao City (Liu, Yang, et al. 2020). Notably, the virus is highly stable at low temperatures. Therefore, it can stay on the surface of cold-chain products or their packaging for a longer time, and can be transferred from one place to another by transportation (Ji et al. 2021; Sun et al. 2021). Since June 2020, the coronavirus was detected on the package of imported frozen products at several places in China, and five local outbreaks of COVID-19 were confirmed to start from such transmission by epidemiological investigation as of December 2020, including those in Beijing in June 2020, Dalian City in July and December 2020, Qingdao City in September 2020, and Tianjin in November 2020 (COVID-19 Field Response Group et al. 2020; Health Commission of Dalian 2020a, 2020b; Liu, Yang, et al. 2020; Pang et al. 2020; Qingdao Municipal Health Commission 2020).

Cold-chain infection cases have rarely been reported from other countries; thus, to what extent the pandemic is due to the human-to-frozen goods-to-human transmission globally remains unclear. But the cases from China confirm that the coronavirus can also spread through trade, especially through cold-chain transportation. Therefore, livestock plants and cold-chain industries should not be neglected for controlling COVID-19 (Ji et al. 2021). The empirical learning from China highlights its importance as an often-overlooked potential coronavirus transmission channel, and its prevention requires national and global coordination and cooperation in effective tracking and disinfection.

We extracted the import trade value of fresh, chilled, or frozen meat and fish of 54 countries/regions in 2016–2020 from the UN Comtrade Database^2^ to illustrate the potential of coronavirus spread through such trade in the global trade network. The dataset contains import information on many categories of traded goods in USD values. We selected five types of the import trade value (Table 1). Total trade value of all commodities in these countries/regions showed an increasing trend before the pandemic, and had a sharp decline in 2020. However, the trade value of fresh, chilled, or frozen meat still increased during the pandemic. Although the trade value of fresh, chilled, or frozen fish declined, its proportion in all commodities was still high. This transnational transportation may add to the risk of human-to-frozen goods-to-human transportation.

**Table 1:** Trade value of imported fresh, chilled, or frozen meat and fish of 54 countries/regions in 2016–2020 (10^9^ USD)

Year	Total trade value of all commodities	Trade value of fresh, chilled, or frozen meat and fish				
		Meat bovine animals (account for %)	Meat of swine (account for %)	Meat of sheep or goats (account for %)	Edible offal of bovine animals, swine, sheep, goats, horses, asses, mules, or hinnies (account for %)	Fish, and fish fillets and other fish meat (whether or not minced) (account for %)
2016	10,982.487	26.274 (0.239)	22.807 (0.208)	3.885 (0.035)	5.298 (0.048)	48.124 (0.438)
2017	12,133.335	28.090 (0.232)	24.225 (0.200)	4.660 (0.038)	5.159 (0.043)	51.571 (0.425)
2018	13,420.287	32.085 (0.239)	23.989 (0.179)	5.387 (0.040)	4.779 (0.036)	56.262 (0.419)
2019	13,018.569	35.381 (0.272)	27.369 (0.210)	5.707 (0.044)	5.344 (0.041)	56.079 (0.431)
2020	12,183.587	36.567 (0.300)	33.109 (0.272)	5.383 (0.044)	5.995 (0.049)	50.633 (0.416)

UN Comtrade Database (https://comtrade.un.org/Data/). The numbers in brackets are the proportion of each type of imported trade value in total trade value of all imported commodities

Available technological solutions, for example, QR codes of goods, can help to enable collaboration. These codes can carry origin information. Scanning QR codes and recording the source and flow information when the goods are shipped offshore and on arrival, along with disinfecting shipped items before sale and register buyer’s information at sale, can facilitate effective prevention of transmission and tracking once transmission is detected. Constructing a network to track the flow of goods between nations needs global cooperation, but its existence would reduce the risk of importing the virus and cut down human-to-frozen goods-to-human transmission.

## Ensuring Adequate Medical Resources and Capacity to Respond Requires Coordination and Cooperation to Share Resources and Experiences

One of the key challenges of coping with the pandemic is to avoid overwhelming the capacity of healthcare systems. Yet, even in the most developed countries, depending on the pattern and pace of an outbreak, a sharp increase of cases could quickly put local and regional healthcare systems under severe stress. In such cases, coordination and cooperation is inevitably required and essential to ensure an adequate medical response.

The Wuhan experience in China is most illustrative. In the early stage of the pandemic in Wuhan City, the local medical system was on the verge of collapse as the virus spread rapidly and the number of confirmed cases soared. Special medical teams were then formed by calling volunteer medical personnel of respiratory and infectious disease departments from other provinces to assist. More than 40,000 front-line medical workers were mobilized from other regions of China to support Wuhan and Hubei Province (Zhang et al. 2020). They also brought vital medical equipment such as ventilators and nucleic acid detectors, which greatly alleviated the pressing equipment shortage of Wuhan and Hubei Province. From this perspective, mutual assistance can be applied between cities, provinces, and countries in a global scale. Bai et al. (2020) called for building a network of assistance in cities to share experience and supplies to enhance the resilience of local institutions and personnel.

Using samples covering 103 countries/regions, our simple correlation analysis suggests statistically significant negative correlation between availability of medical resources—represented by hospital beds per 1,000 population and medical doctors per 10,000 population—and case fatality rate^3^ (Pearson’s correlation −0.197 and −0.209, *P* < 0.05, *n* = 103). Data for density of hospital beds during 2016–2019 in the 103 countries/regions were obtained from the Organization for Economic Co-operation and Development (OECD)^4^ and the World Health Organization (WHO)^5^; and data for density of medical doctors during 2010–2018 in the 103 countries/regions were obtained from *World Health Statistics 2020* (WHO 2020).

## Concluding Remarks: A Global Network Promoting “Dual Health Paradigm” can Enhance Human Health Risk Resilience

The probability of new disease outbreaks is increasing due to human activity (Arora and Mishra 2020). In addition to the current COVID-19 pandemic, there have been several global pandemics this century, including the SARS (Severe Acute Respiratory Syndrome Coronavirus), MERS (Middle East Respiratory Syndrome), and Ebola pandemics. Similar but different in scale, the COVID-19 pandemic reveals some long-recognized health risk challenges as well as some emerging characteristics that are critical for long-term societal resilience building against those seemingly growing global health risks.

From a hazard perspective, the COVID-19 pandemic has again illustrated the consequential impacts of human exploitation of natural resources on human health (Fiorella et al. 2020), fundamentally the imbalance between “Earth health” and human health. In the context of sustainable natural resources management and ecosystem restoration, back in 2004, we called for a Dual Health Model that combines Earth health and human-health (Shi et al. 2004). Today, we argue that a similar dual health model is also vital in preventing global pandemics.

From an “Earth health” perspective, we see a range of global health risk challenges that are the consequences of human actions: rising global average temperature due to continuous consumption of fossil fuels and greenhouse gas emissions; the accelerating rate of extinction of marine species and the degradation of marine ecosystems due to overfishing and the loss of biomes; deforestation, grassland degradation, wetland shrinkage, and desertification due to rapid urbanization and agricultural expansion, just to name a few. The biogeochemical processes of the Earth’s ecosystem are closely related to the vital geochemical process of the world’s human system. Changes in the ecosystem also affect the rhythm of metabolism, material circulation, energy flow, and other vital geochemical processes of the human system. Once harmful substances or viruses enter the human health system through the production chain, supply chain, and food chain, they could lead to diseases and other damages. To break the vicious circle, protecting human health requires the protection of Earth ecosystem stability and improvement of its service capacity, that is, preservation of “Earth health.” As we achieve a healthy Earth, we could also reduce the occurrences of infectious diseases, which in turn protects human health. As a global disaster, the COVID-19 pandemic once again highlights the importance of the root causes of the human vulnerability to disaster risks—inequality, poverty, and exclusion. Without addressing those fundamental underlying causes, those most affected will see their risks increase (Alcántara-Ayala et al. 2020). A latest model estimation by the *Economist* suggests that COVID-19 has claimed 7.1–12.7 million lives worldwide, which is more than three times of the official count (Economist 2021a). Not surprisingly, while the rich world suffered relatively badly this time, still, the overwhelming majority of the 6.7 million or so uncounted deaths have been in the poor and middle-income countries (Economist 2021b). The current COVID-19 pandemic has also made painfully clear the remarkable inadequacy of global mechanisms for collective action and coordinated response. Implementing the three example lessons cited in this short article—swift and strong government-led response, prevention of transmission through cold-chain food linkages, and secure access to adequate medical resources—all demand coordination and cooperation locally, regionally, and globally.

The ongoing COVID-19 vaccination campaign is another example that highlights the crucial importance of global cooperation as well as the danger of not cooperating. On the one hand, the successful development of a variety of vaccines in a record speed is the result of collaborations on all fronts, among scientists around the world and between companies and governments across different countries. On the other hand, now the fair and effective vaccination equally demands global coordination and cooperation, from production and distribution to eventual inoculation. While the rich part of the world is quickly being vaccinated, all agree that until vaccine supplies also reach the poorer countries, the world would not be COVID-free.

Overall the on-going COVID-19 pandemic disaster serves as another reminder of the growing systemic risks faced by an increasingly interconnected and interdependent world. In such a world, no one is safe until all are safe. Enhancing global health risk resilience thus requires us to “build a global community of health for all,” as called for by President Xi of China in his recent remarks at the G20 Global Heath Summit on 21 May 2021. This address included five action points: (1) put people and their lives first; (2) follow science-based policies and ensure a coordinated and systemic response; (3) stick together and promote solidarity and cooperation; (4) uphold fairness and equity as we strive to close the immunization gap; and (5) address both the symptoms and root causes of the pandemic as we work to improve the governance system.^6^

It is in this context of building a global community of health for all that we are calling for a global network to enhance integrated human health risk resilience. This increased capacity can facilitate coordination and cooperation at all levels, and promote a “one health” paradigm shift to fundamentally reduce global environmental and human health risks.

Many are calling for building back better, an aspiration linked to the societal and development transition towards low to zero-carbon development, among other objectives. In that sense, the pandemic could be a catalyst, or as the World Economic Forum calls it, The Great Reset, for the urgently needed acceleration of the transformational changes required for an inclusive, resilient, and sustainable future world for all. A global network for integrated human health resilience would be conducive to such a pursuit.

## Footnotes

1 Daily growth rate of new infection cases is the number of confirmed cases in the day of concern minus the number of confirmed cases in the previous day, divided by the latter.

2 https://comtrade.un.org/Data/.

3 Case fatality rate (‰) is the ratio of total deaths to total cumulative number of confirmed cases.

4 https://stats.oecd.org/Index.aspx#.

5 https://www.who.int/.

6 https://www.fmprc.gov.cn/mfa_eng/zxxx_662805/t1877666.shtml.
